# Serum and Urinary Selenium Status in Patients with the Pre-diabetes and Diabetes in Northeast China

**DOI:** 10.1007/s12011-018-1604-7

**Published:** 2018-12-14

**Authors:** Qi Zhou, Wenjia Guo, Yanan Jia, Jiancheng Xu

**Affiliations:** 1 Department of Pediatrics, First Hospital of Jilin University, Changchun, 130021 China; 2 Department of Laboratory Medicine, First Hospital of Jilin University, Changchun, 130021 China

**Keywords:** Selenium, Impaired fasting glucose, Impaired glucose tolerance, Type 1 diabetes, Type 2 diabetes

## Abstract

Homeostasis imbalance of selenium (Se) in diabetes has received great attention. This study investigated serum and urinary Se levels in patients with impaired fasting glucose (IFG), impaired glucose tolerance (IGT), type 1 diabetes (T1D), and type 2 diabetes (T2D) in Northeast Chinese populations. From January 2010 to October 2011, patients with IFG (*n* = 12), IGT (*n* = 15), T1D (*n* = 25), T2D (*n* = 137), and healthy controls (*n* = 50) were enrolled in the First Hospital of Jilin University. Se was detected using inductively coupled plasma spectrometer. The serum Se level was dramatically lower in patients with T1D and was significantly higher in IFG subjects, and the urinary Se concentration was markedly lower in IGT and T2D groups. The serum Se levels were positively correlated with serum zinc (Zn) in both IFG and IGT groups, while urinary Se were positively associated with urinary Zn and copper (Cu) in IGT group. The serum Se levels were positively correlated with serum Cu in both T1D and T2D groups, and urinary levels of Se were positively associated with serum zinc and urinary Cu, Zn, calcium (Ca), and magnesium (Mg) and negatively correlated with serum Ca and Mg in T2D group, while the urinary levels of Se were positively correlated with urinary Zn and Mg both in peripheral neuropathy (DPN) and retinopathy (DR) groups. One month of simvastatin therapy reduced serum Se levels. These results suggest the potential role of Se in diabetes should receive attention.

## Introduction

Diabetes mellitus (DM) is a heterogeneous group of clinical and genetic metabolic disorders recognized by an abnormally high level of glucose in the blood. It is classified broadly into two types—type 1 diabetes (T1D) and type 2 diabetes (T2D). According to the latest report of the International Diabetes Federation Diabetes Atlas, the overall prevalence of diabetes in adults is 9.1%, implying that 415 million adults suffer from diabetes globally [1]. Moreover, 318 million adults have impaired glucose regulation and are at a high risk for developing diabetes in the future [1]. China ranks number one, with the highest number of people with diabetes [1]. Both types of DM can cause microvascular complications, e.g., diabetic nephropathy (DN), peripheral neuropathy (DPN), and retinopathy (DR) [2].

Our previous studies have shown that whole-body level dysregulation of copper, zinc, calcium, and magnesium plays an important role in the pathogenesis of diabetes and its complications [3–6]. As an essential trace element, selenium (Se) has a major metabolic significance for human beings. Se is a basic component of selenoprotein which plays a functional role in redox homeostasis, thyroid hormone metabolism, and protection from oxidative stress and inflammation [7]. Some of the body of Se are derived from foods, and some of which are products of metabolism. Most Se-enriched foods are natural foods such as organ meat, seafood, cereals, and crops. The level of Se intake varies greatly among individuals and populations. The average estimated Se intake based on the Chinese Food Pagoda (EI_CHFP_) in 12 provinces from the 4th China Total Diet Study, within the range of 66.23–145.20 μg/day, was greater than the China recommended nutrient intake (RNI) (60 μg/day) [8]. The average estimated dietary Se intakes (EI_TDS_) in China (88 μg/day) was in line with its range of EI_CHFP_ (81.01–124.25 μg/day), but that in half of the regions failed to achieve their lowest EI_CHFP_ [8].

The association between Se and diabetes is in fact very complicated. Although numerous epidemiological studies have explored this association, their findings have been inconclusive. Some researchers suggest that high Se level could reduce the prevalence of diabetes [9], whereas others conclude that a high level of serum Se could be related to the increased prevalence of diabetes [7, 10–12]. Recently, some studies suggest that Se supplementation in patients with T2D may be associated with adverse effects on blood glucose homeostasis [13, 14]. Therefore, the inconsistent study of the relationship between Se and diabetes may be related to the population, and may also be related to the detection method.

Most previous studies focused on the comparison of serum Se in diabetic and non-diabetic patients. However, there are few studies about the effect of serum Se levels on pre-diabetes and diabetes with or without complications in the Chinese population. Therefore, we detected the serum and urinary Se levels in patients with impaired fasting glucose (IFG), impaired glucose tolerance (IGT), type 1 diabetes (T1D), and type 2 diabetes (T2D) in Northeast China (Mainly from Jilin, Liaoning, and Heilongjiang provinces, where the average temperature seems to be the lowest across the whole country).

## Research Design and Methods

### Ethics Statement

This study was approved by the institutional ethics committee of the First Hospital of Jilin University. Written informed consent was obtained from all subjects. For the children who were younger than 18 years old, their parents provided written informed consent and when possible, child subjects provided written assent. All data were obtained from the electronic medical records of our hospital, and the patients were anonymized.

### Subjects

The population in this study is detailed elsewhere [3]. One hundred eighty nine patients with diabetes or pre-diabetes and 50 healthy controls were recruited during the period from January 2010 to October 2011 in the present study. Briefly, 31 healthy male and 19 healthy female participants between the ages of 20 and 59 years old with a median age of 50 years old, and who were with fasting serum glucose< 7 mmol/L, were eligible. The patients enrolled are as follows: 12 patients with impaired fasting glucose (IFG, 8 males and 4 females, ages from 31 to 53), 15 patients with impaired glucose tolerance (IGT, 9 males and 6 females, ages from 40 to 56), 25 patients with type 1 diabetes (T1D, 8 males and 17 females, ages from 9 to 33 with a median age of 25 years old), and 137 patients with T2D (85 males and 52 females, ages from 42 to 62 with a median age of 56 years old). T2D patients with complications are as follows: 24 patients with nephropathy (DN, 19 males and 5 females, ages from 28 to 84 with a median age of 60 years old), and 34 patients with DR (15 males and 19 females with an ages range of 29–74 years at median age of 60 years old), and 50 patients with peripheral neuropathy (DPN, 29 males and 21 females, ages range of 27–79 with a median age of 56 years old).

All demographic data for these patients were obtained from the electronic medical records of our hospital. Body mass index (BMI) was calculated as weight in kilograms divided by the square of height in meters (kg/m^2^). The diagnosis of impaired fasting glucose (IFG) was based on fasting serum glucose level (6.1–6.9 mmol/L) and non-fasting serum glucose level (< 7.8 mmol/L). Impaired glucose tolerance (IGT) was defined by fasting glucose < 7.0 mmol/L, but not non-fasting glucose level in 7.8–11.0 mmol/L. The diagnosis of diabetes was based on fasting serum glucose level (≥ 7.0 mmol/L), 2-h serum glucose level (≥ 11.1 mmol/L), and hemoglobin A1c (HbA1c) value (≥ 6.5%).

### Laboratory Measurements

Glucose, blood urea nitrogen (BUN), creatinine, total cholesterol (CHO), triglyceride (TG), high-density lipoprotein (HLDL), and low-density lipoprotein cholesterol (LDL) were performed on Hitachi 7600-210 automatic analyzer (Hitachi High-Technologies, Tokyo, Japan). HbA1c were detected on analyzer (BioRad, Hemel Hempstead, UK). Red blood cell (RBC) and hemoglobin were measured on Sysmex XN-series system (Sysmex, Kobe, Japan). Se was detected using inductively coupled plasma spectrometer (ICP-MS, PerkinElmer Life and Analytical Sciences, Inc., CT, USA).

Before blood collection in the morning, each participant fasted for at least 8 h overnight. Samples were collected in commercial tubes for analysis of laboratory parameters, and into special metal-free tubes (Polyethylene centrifugal pipe, Shenhua experimental equipment sales department, Haimen, pre-soaked in 10% *v*/*v* nitric acid for 48 h) for analysis of trace element. Blood was centrifuged, separated, and aliquoted within 2 h of collection. Excluding unqualified samples which were hemolysis, lipidemia, and jaundice, all qualified samples were sent to the laboratory for examination as soon as possible. For the detection of trace element, 0.5 ml serum was digested in 2 ml 10% nitric acid at room temperature for 30 min. Subsequently, serum was digested at 100 °C for 2 h. Deionized water was added to each sample to increase the volume to 15 ml. A 24-h urine sample was obtained after admission to measure eGFR. The eGFR was calculated by Cockroft-Gault equation: creatinine clearance (Ccr) = ((140 − age) × body weight)/(serum creatinine × 72) × (0.85, if female) [15].

### Comparison of Se Levels in the Patients with T2D Before and After 1-Month Treatment with Simvastatin

The population in this study is detailed elsewhere [3]. We enrolled 24 patients with T2D who were not originally with any lipid-lowering drug. Inclusion criteria were CHO > 6.22 mmol/L and LDL > 4.14 mmol/L. Exclusion criteria were treatment with lipid-lowering drugs. Patients were asked to take simvastatin with 10 mg/day for 1 month. Blood were collected into commercial tubes and special metal-free tubes from the fasted patients at the beginning and the end of the 1 month treatment.

### Statistical Analysis

Statistical analysis was performed with SPSS 21.0 (IBM) software. Continuous variables were expressed as median (interquartile range) and categorical variables as number (percent). The data were analyzed using the chi-square test or Fisher’s exact test for categorical variables, or with the Mann-Whitney *U* test for continuous variables. Spearman rank correlation analysis was used to evaluate the correlations. Baseline characteristics were adjusted for age, sex, BMI, hypertension, and dyslipidemia by analysis of covariance using general linear models. A two-tailed *P* value of less than or equal to 0.05 was considered as statistically significant.

## Results

### Study Subjects and Baseline Characteristics

**Fig. 1:**
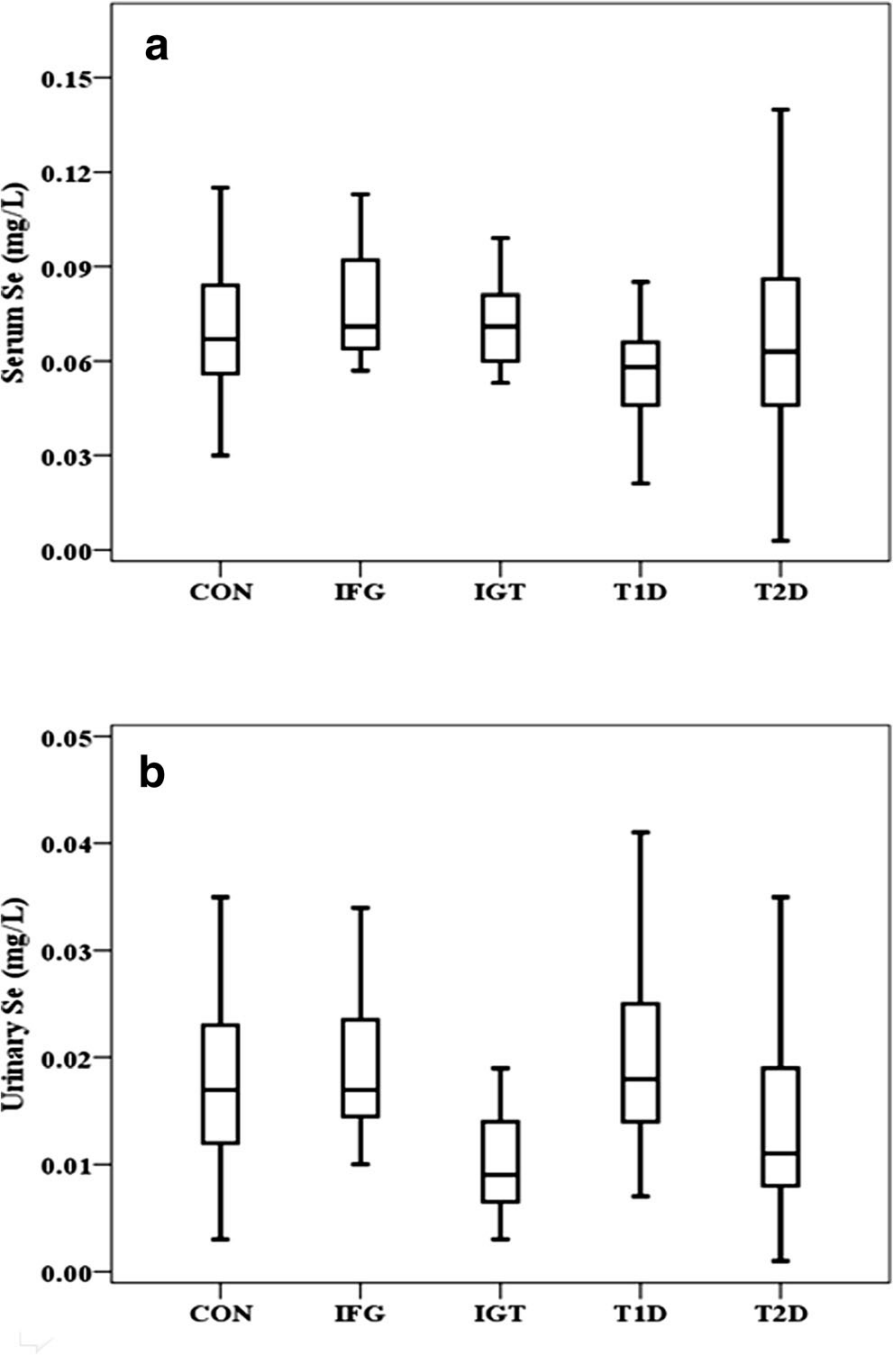
Levels of Se in healthy control, IFG, IGT, T1D, and T2D groups. Boxplots display the extremes, the upper and lower quartiles, and the median of the maximum difference in healthy controls, IFG, IGT, and T2D groups. The median for each dataset is indicated by the centerline, and the first and third quartiles are presented by the edges of the area, which is known as the interquartile range (IQR). *P* value of serum Se levels was < 0.05 between healthy control group and IFG, IGT, T1D, and T2D groups (**a**). *P* value of urinary Se levels was < 0.05 between healthy control group and IFG, IGT, T1D, and T2D groups (**b**)

Baseline characteristics of study subjects were detailed in the previously published paper [4]. Compared to control groups, the serum Se level was dramatically lower in patients with T1D and was significantly higher in IFG subjects, while it was not markedly changed in both IGT and T2D groups (Table 1, Fig. 1a). The urinary Se concentration was markedly lower in IGT and T2D groups but not different in IFG and T1D subjects (Table 1, Fig. 1b).

**Table 1:** Baseline characteristics of subjects

	CON (*n* = 50)	IFG (*n* = 12)	*P*	IGT (*n* = 15)	*P*	T1D (*n* = 25)	*P*	T2D (*n* = 137)	*P*
Cu (mg/L)	0.8 (0.7–0.99)	1.13 (1.02–1.29)	0.001*	1.15 (1.03–1.24)	< 0.001*	0.94 (0.75–1.19)	0.352	1.07 (0.95–1.33)	< 0.001*
UCu (μg/L)	25 (22–40)	32 (28.3–42.5)	0.293	26 (24–28)	0.306	25 (23–27.5)	0.187	30 (25.5–40.5)	0.408
Zn (mg/L)	0.81 (0.67–0.93)	0.75 (0.7–0.84)	0.462	0.77 (0.67–0.87)	0.834	0.59 (0.53–0.75)	0.056	0.61 (0.51–0.75)	< 0.001*
UZn (mg/L)	0.2 (0.14–0.32)	0.32 (0.26–0.37)	0.745	0.27 (0.19–0.41)	0.836	0.86 (0.67–0.91)	< 0.001*	0.48 (0.38–0.57)	< 0.001*
Zn/Cu	0.92 (0.84–1.07)	0.68 (0.62–0.73)	0.009*	0.65 (0.62–0.77)	0.002*	0.64 (0.57–0.77)	0.018*	0.56 (0.43–0.68)	<0.001*
Mg (mg/L)	35.7 (30.8–39.7)	21.6 (17.4–25.7)	< 0.001*	21.4 (20.5–26.8)	< 0.001*	15.4 (12.2–17.7)	< 0.001*	21.6 (16.4–27)	< 0.001*
UMg (mg/L)	34.4 (15.1–47.3)	22.9 (14–71)	0.446	20.1 (10.7–42.1)	0.231	57.8 (46.4–65.7)	0.86	50.2 (40.1–77)	0.159
Ca (mg/L)	165 (145.8–177.2)	47.4 (25.9–52.9)	< 0.001*	130.4 (111.2–133.3)	< 0.001*	97.3 (77.7–111)	0.007*	95 (62.9–131)	< 0.001*
UCa (mg/L)	63.3 (31–94.1)	123.6 (64.1–147.1)	0.027*	39.1 (16.1–75.1)	0.133	104.5 (44.4–169.7)	0.208	85.9 (39.1–137.8)	0.099
Se (mg/L)	0.067(0.056–0.084)	0.071(0.064–0.103)	0.021*	0.071(0.059–0.082)	0.791	0.058(0.058–0.07)	0.008*	0.063(0.045–0.086)	0.112
Use (mg/L)	0.017(0.012–0.023)	0.017(0.014–0.024)	0.544	0.009(0.006–0.017)	0.006*	0.018(0.014–0.025)	0.319	0.011(0.008–0.019)	0.007*

Data are presented as number (%) or median (interquartile range). *Cu* serum copper, *UCu* urinary copper, *Zn* serum zinc, *UZn* urinary zinc, *Mg* serum magnesium, *Umg* urinary magnesium, *Ca* serum calcium, *UCa* urinary calcium, *Se* serum selenium, *USe* urinary selenium

**Fig. 2:**
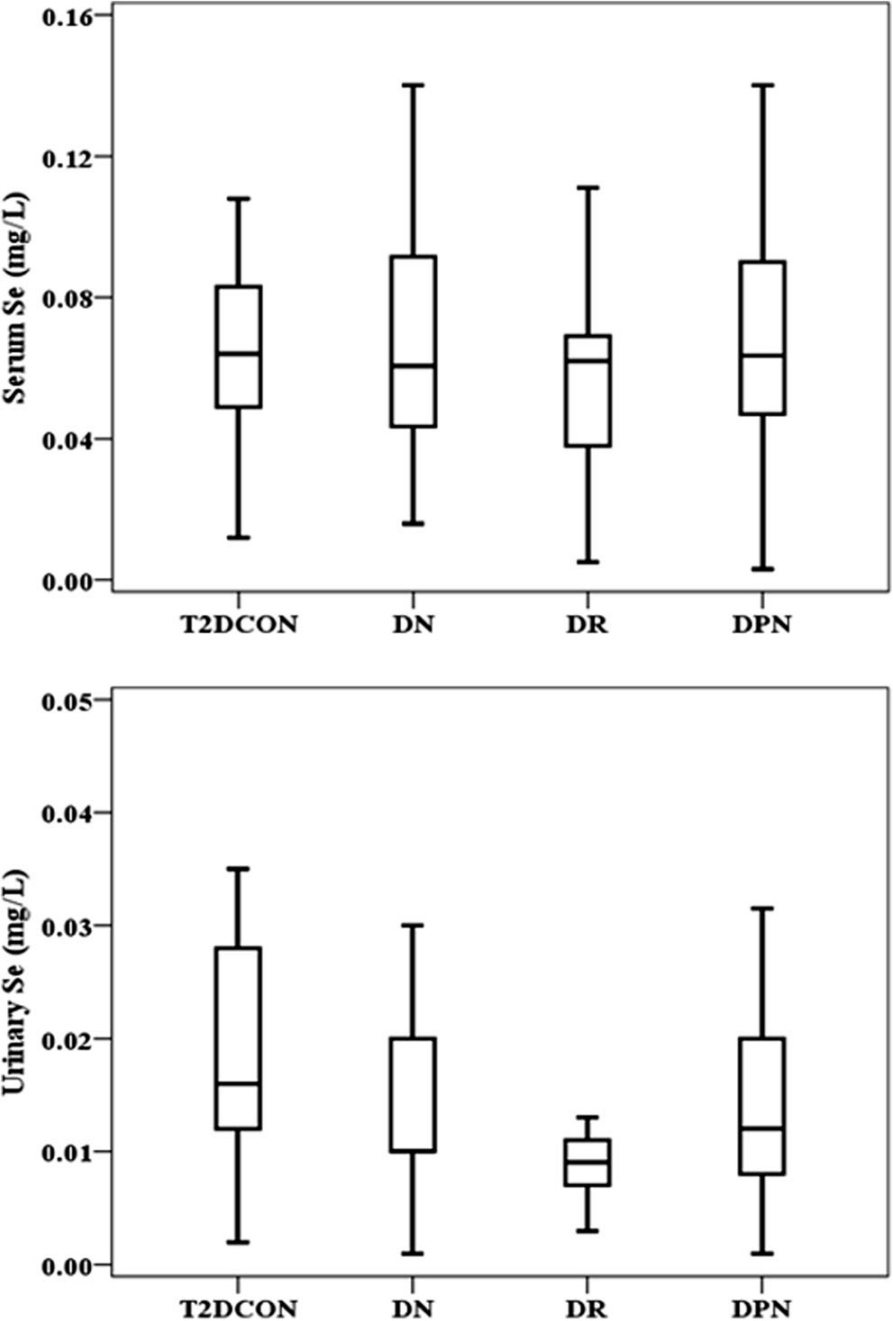
Levels of Se in T2D without complications, DN, DR, and DPN groups. *P* value of serum Se levels was < 0.05 between T2D without complications (T2DCON), DN, DR, and DPN groups (**a**). *P* value of urinary Se levels was < 0.05 between T2D without complications (T2DCON), DN, DR, and DPN groups (**b**)

Baseline characteristics of T2D subjects were detailed in the previously published paper [4]. Considering T2D groups including those who had been diagnosed with DN, DR, or DPN, the serum and urinary Se levels in those who had been diagnosed with complications were further analyzed. Compared to uncomplicated T2D subjects, the serum Se levels were not statistically changed in all T2D patients with complications (Table 2, Fig. 2a), while the urinary Se levels were all significantly lower in T2D patients with DN, DR, or DPN (Table 2, Fig. 2b).

**Table 2:** Baseline characteristics of T2D subjects

	T2D Con (*n* = 29)	DN (*n* = 24)	*P*	DR (*n* = 34)	*P*	DPN (*n* = 50)	*P*
Cu (mg/L)	1 (0.94–1.15)	1.26 (1.07–1.42)	0.015*	1.05 (0.92–1.48)	0.717	1.08 (0.92–1.38)	0.298
UCu (μg/L)	29 (24.5–36.5)	37 (28.3–49)	0.947	28.5 (26.8–36)	0.274	32 (22.8–43)	0.815
Zn (mg/L)	0.73 (0.55–0.79)	0.59 (0.48–0.76)	0.157	0.58 (0.46–0.63)	0.002*	0.63 (0.59–0.75)	0.08
UZn (mg/L)	0.47 (0.28–0.53)	0.44 (0.3–0.52)	0.685	0.45 (0.25–0.52)	0.824	0.52 (0.44–0.63)	< 0.001*
Zn/Cu	0.66 (0.55–0.76)	0.5 (0.36–0.56)	0.006*	0.48 (0.4–0.64)	0.012*	0.58 (0.46–0.68)	0.111
Mg (mg/L)	18.6 (15.9–24.5)	16.4 (12.6–25.7)	0.052	23.8 (21.6–28.7)	0.002*	21.6 (15.8–28.6)	0.447
UMg (mg/L)	47.7 (39.4–67.1)	48.2 (39.1–87.7)	0.711	46.4 (37.3–74.1)	0.699	53.5 (42.8–77.5)	0.229
Ca (mg/L)	98.5 (78.9–114.2)	66.8 (55.7–127.7)	0.043*	105.7 (70.9–138.9)	0.832	93.8 (56.7–136.6)	0.853
UCa (mg/L)	84.1 (59.5–138.8)	51.8 (12.7–160.3)	0.646	66.5 (35.9–116.2)	0.256	92.7 (63.7–127)	0.827
Se (mg/L)	0.064(0.048–0.084)	0.061(0.042–0.094)	0.775	0.062(0.038–0.069)	0.243	0.064 (0.046–0.09)	0.776
Use (mg/L)	0.016(0.012–0.03)	0.01(0.01–0.02)	0.033*	0.009(0.007–0.011)	< 0.001*	0.012(0.008–0.02)	0.038*

Data presentation and abbreviations’ spelling are the same as the description for Table 1

^*^P < 0.05 vs T2D control group

### Correlations Between Serum or Urinary Se and Laboratory Parameters in Groups

Presumed that serum Se is related to certain laboratory parameters, we further individually analyze associations between serum or urinary Se and other laboratory parameters among the group of control, IFG, IGT, T1D, and T2D (Table 3).

**Table 3:** Associations between serum Se or urinary Se level as a continuous variable and laboratory parameters in subjects

	CON (*n* = 50)				IFG (*n* = 12)				IGT (*n* = 15)				T1D (*n* = 25)				T2D (*n* = 137)			
	Se		USe		Se		USe		Se		USe		Se		USe		Se		USe	
	*r*	*P*	*r*	*P*	*r*	*P*	*r*	*P*	*r*	*P*	*r*	*P*	*r*	*P*	*r*	*P*	*r*	*P*	*r*	*P*
Glu	− 0.216	0.131	− 0.084	0.561	0.206	0.52	0.249	0.436	0.112	0.691	0.077	0.786	− 0.121	0.563	0.098	0.641	0.052	0.544	0.051	0.553
HbA1c	− 0.277	0.051	− 0.107	0.462	0.163	0.613	0.108	0.738	0.148	0.599	0.351	0.2	− 0.106	0.615	0.306	0.137	0.104	0.229	− 0.071	0.407
RBC	− 0.017	0.909	− 0.072	0.62	− 0.238	0.456	0.249	0.435	0.349	0.202	0.115	0.683	0.103	0.623	0.128	0.543	0.036	0.674	0.366	< 0.001*
Hb	− 0.064	0.659	− 0.001	0.994	− 0.147	0.649	0.206	0.521	0.325	0.237	0.179	0.522	0.112	0.593	0.124	0.554	0.035	0.687	0.309	< 0.001*
Cu	0.447	0.001*	0.004	0.981	− 0.165	0.608	− 0.368	0.239	0.284	0.305	0.169	0.548	0.455	0.022*	0.041	0.846	0.266	0.002*	− 0.012	0.891
UCu	− 0.083	0.565	0.193	0.179	0.269	0.398	− 0.392	0.208	0.014	0.962	0.573	0.026*	0.377	0.064	0.185	0.375	0.17	0.047	0.226	0.008*
Zn	0.352	0.012*	− 0.277	0.051	0.689	0.013*	0.069	0.832	0.517	0.049*	− 0.145	0.605	0.179	0.393	− 0.041	0.846	0.135	0.117	0.212	0.013*
UZn	− 0.036	0.806	0.189	0.188	0.613	0.034*	− 0.109	0.736	0.021	0.94	0.615	0.015*	− 0.09	0.667	0.06	0.777	0.099	0.251	0.259	0.002*
Ca	0.142	0.325	− 0.062	0.669	− 0.344	0.273	0.172	0.594	0.463	0.082	− 0.342	0.212	− 0.028	0.894	0.216	0.299	0.058	0.498	− 0.185	0.03*
UCa	− 0.046	0.752	0.599	< 0.001*	0.271	0.394	− 0.108	0.737	− 0.038	0.894	0.152	0.587	− 0.147	0.483	− 0.013	0.949	0.019	0.822	0.272	0.001*
Mg	0.027	0.85	− 0.142	0.326	− 0.103	0.751	− 0.218	0.497	0.643	0.01*	− 0.016	0.954	− 0.106	0.614	0.187	0.372	0.046	0.595	− 0.292	0.001*
UMg	− 0.022	0.881	0.43	0.002*	− 0.007	0.982	0.112	0.728	− 0.041	0.884	0.447	0.095	− 0.009	0.965	− 0.187	0.371	0.117	0.174	0.326	< 0.001*
BUN	0.15	0.3	− 0.042	0.771	0.156	0.628	− 0.33	0.294	0.469	0.078	0.148	0.6	− 0.271	0.19	0.155	0.459	0.112	0.193	0.031	0.722
Cre	0.214	0.136	0.071	0.625	− 0.294	0.354	− 0.223	0.486	0.678	0.005*	− 0.061	0.828	0.27	0.191	0.05	0.813	0.035	0.684	− 0.045	0.602
eGFR	0.142	0.324	0.029	0.842	0.141	0.661	0.098	0.761	− 0.282	0.309	− 0.137	0.625	− 0.091	0.664	− 0.04	0.851	− 0.049	0.569	0.154	0.073
CHO	− 0.018	0.901	0.009	0.953	0.418	0.177	0.262	0.41	0.33	0.23	0.057	0.841	− 0.104	0.622	0.045	0.829	− 0.074	0.389	− 0.133	0.122
TG	0.073	0.613	0.042	0.771	0.331	0.293	− 0.033	0.92	0.521	0.046*	− 0.069	0.807	0.197	0.344	− 0.069	0.743	− 0.019	0.824	− 0.032	0.711
HDL	− 0.078	0.59	− 0.013	0.929	− 0.405	0.191	− 0.329	0.296	0.103	0.715	− 0.187	0.505	− 0.236	0.256	− 0.18	0.39	− 0.048	0.576	− 0.134	0.118
LDL	− 0.084	0.56	− 0.16	0.268	0.22	0.493	0.226	0.48	0.08	0.778	− 0.227	0.417	− 0.212	0.308	0.227	0.275	− 0.029	0.736	− 0.136	0.112
USe	− 0.023	0.873	–	——.	− 0.044	0.892	–	——.	0.009	0.975	–	——.	0.044	0.836	–	——.	0.127	0.139	–	——.

Data presentation and abbreviations’ spelling are the same as the description for Table 1

^*^P < 0.05 for the association

In control group, serum levels of Se were positively correlated with serum Cu and Zn levels, while urinary levels of Se were positively associated with urinary Ca and Mg. In IFG group, serum Se levels were positively correlated with serum and urinary Zn. In IGT group, serum Se levels were positively correlated with serum Zn, Mg, Cre, and TG, while urinary Se were positively associated with urinary Cu and Zn. In T1D group, serum Se levels were positively correlated with serum Cu. In T2D group, serum levels of Se were positively correlated with serum and urinary Cu levels, while urinary levels of Se were positively associated with RBC count, Hb, serum Zn, and urinary Cu, Zn, Ca, and Mg and negatively correlated with serum Ca and Mg.

### Correlations Between Serum or Urinary Se and Laboratory Parameters inT2D With or Without Complications

Since the above analysis showed that serum or urinary Se is related to certain laboratory indicators among the group of control, IFG, IGT, T1D, and T2D, we further analyzed the associations in T2D with or without complications (Table 4).

**Table 4:** Associations between serum Se or urinary Se level as a continuous variable and laboratory parameters in T2D subjects

	T2D Con (*n* = 29)				DN (*n* = 24)				DR (*n* = 34)				DPN (*n* = 50)			
	Se		USe		Se		USe		Se		USe		Se		USe	
	r	*P*	*r*	*P*	*r*	*P*	*r*	*P*	*r*	*P*	*r*	*P*	*r*	*P*	*r*	*P*
Glu	− 0.223	0.244	0.099	0.609	0.453	0.026*	0.03	0.888	− 0.168	0.342	− 0.189	0.284	0.092	0.524	− 0.025	0.862
HbA1c	− 0.141	0.466	− 0.148	0.444	0.096	0.656	0.052	0.811	− 0.04	0.824	− 0.227	0.197	0.253	0.076	− 0.006	0.97
RBC	0.014	0.941	0.15	0.438	0.427	0.038*	0.401	0.052	− 0.33	0.056	0.469	0.005*	− 0.07	0.628	0.189	0.19
Hb	0.224	0.243	0.07	0.718	0.404	0.05	0.407	0.048*	− 0.358	0.038*	0.562	0.001*	− 0.047	0.745	0.141	0.33
Cu	0.08	0.682	− 0.382	0.041*	− 0.003	0.987	0.058	0.788	0.279	0.11	0.022	0.904	0.41	0.003*	0.204	0.155
UCu	0.407	0.028*	0.281	0.139	0.389	0.06	0.162	0.45	0.077	0.663	− 0.111	0.53	0.076	0.601	0.329	0.02*
Zn	0.068	0.727	− 0.02	0.917	− 0.166	0.437	0.118	0.581	0.184	0.297	0.209	0.235	0.187	0.192	0.155	0.283
UZn	0.242	0.206	0.184	0.34	0.343	0.101	0.267	0.208	− 0.162	0.36	0.369	0.032*	− 0.028	0.849	0.321	0.023*
Ca	0.163	0.398	− 0.198	0.303	− 0.101	0.639	0.038	0.859	0.302	0.083	0.169	0.338	− 0.028	0.845	− 0.365	0.009*
UCa	0.111	0.566	0.254	0.184	0.024	0.913	0.438	0.032*	− 0.053	0.766	0.117	0.511	− 0.004	0.98	0.212	0.14
Mg	0.267	0.162	− 0.301	0.113	− 0.039	0.857	0.053	0.805	0.254	0.148	− 0.083	0.642	− 0.017	0.906	− 0.312	0.028*
UMg	0.417	0.025*	0.273	0.152	0.081	0.705	0.402	0.051	− 0.228	0.194	0.446	0.008*	0.116	0.421	0.295	0.038*
BUN	0.126	0.515	0.315	0.096	0.073	0.735	0.045	0.834	0.207	0.24	0.044	0.803	0.205	0.154	0.051	0.726
Cre	0.13	0.501	0.195	0.312	− 0.159	0.459	0.096	0.657	0.205	0.245	− 0.115	0.516	− 0.022	0.88	− 0.029	0.839
eGFR	− 0.001	0.996	0.112	0.564	0.203	0.341	0.292	0.166	− 0.222	0.207	0.233	0.185	− 0.015	0.92	0.114	0.431
CHO	0.208	0.28	− 0.093	0.631	− 0.047	0.827	0.044	0.837	− 0.035	0.846	− 0.047	0.794	− 0.23	0.109	− 0.238	0.097
TG	0.112	0.563	− 0.079	0.684	0.041	0.85	− 0.003	0.987	− 0.107	0.548	− 0.019	0.914	− 0.052	0.721	− 0.087	0.547
HDL	− 0.093	0.632	− 0.045	0.816	0.039	0.858	− 0.265	0.21	0.03	0.866	0.08	0.654	− 0.157	0.278	− 0.121	0.402
LDL	0.122	0.529	− 0.14	0.469	− 0.142	0.508	− 0.027	0.901	0.11	0.534	− 0.183	0.301	− 0.156	0.281	− 0.145	0.314
USe	− 0.152	0.431	–	——.	0.53	0.008*	–	——.	− 0.216	0.221	–	——.	0.104	0.471	–	——.

Data presentation and abbreviations’ spellings are the same as the description for Table 1

^*^P < 0.05 for the association

In uncomplicated T2D subjects, serum levels of Se were positively correlated with urinary Cu and Mg levels, while urinary levels of Se were negatively associated with urinary Cu and Mg. In DN group, serum levels of Se were positively correlated with RBC count, Hb, serum GLU, and urinary Se, while urinary Se were positively associated with Hb, urinary Ca, and serum Se. In DR group, serum levels of Se were negatively correlated with Hb and urinary Se were positively associated with RBC count, Hb, and urinary Zn and Mg. In DPN group, serum levels of Se were positively correlated with serum Cu levels, while urinary levels of Se were positively associated with urinary Cu, Zn, and Mg and negatively correlated with serum Ca and Mg.

### Effect of Simvastatin Treatment on Serum or Urinary Se Levels and Other Laboratory Parameters in Patients with T2D

Statins treatment was often used to lower lipid in patients with T2D. Therefore, the effect of simvastatin therapy for 1 month on serum or urinary Se levels was detected in patients with T2D. Therapy with simvastatin for 1 month markedly reduced serum CHO and LDL levels as expected [3]. One month of simvastatin therapy reduced serum Se levels [0.049(0.031–0.069) mg/L vs 0.062(0.060–0.090) mg/L), *P* < 0.05], but had no effects on urinary Se levels [0.019(0.008–0.028) mg/L vs 0.012(0.008–0.025) mg/L, *P* > 0.05].

## Discussion

### Serum and Urinary Se Levels in Pre-diabetes and Diabetes

Research about the association between serum Se levels and diabetes has been widely reported [12, 14]. In this study, the serum Se level was dramatically lower in patients with T1D and was significantly higher in IFG subjects. Our results validated recent studies that low serum Se was lower in children with T1D in Egypt [16] and high serum Se levels were associated with IFG in Linyi, China [17]. The association of urinary Se with diabetes was still controversial. A Chinese research indicated that the levels of urinary Se were significantly higher in T2D groups [18], whereas another Spanish research concluded that mean urinary Se concentration in diabetic patients was not significantly different from those measured in the control group [9]. Our results indicated that the urinary Se concentration was markedly lower in IGT and T2D groups. The possible reason for the inconsistency between our findings and previous studies is that populations from different regions have different eating habits. Our study showed the urinary Se levels were all significantly lower in T2D patients with DN, DR, or DPN than uncomplicated T2D subjects. To our knowledge, this is the first study comparing Se in uncomplicated diabetes and diabetic complications.

### Correlations Between Serum or Urinary Se and Laboratory Parameters in Groups

Our study explored the association of Se with other trace elements in diabetes. To our knowledge, we demonstrated for the first time that the serum Se levels were positively correlated with serum Zn in both IFG and IGT groups, while urinary Se were positively associated with urinary Zn and Cu in IGT group. Previous studies showed urinary Se were associated with altered FPG, IFG, or diabetes risk [19] and hair Se were positively correlated with blood glucose levels [20]. Unlike previous studies [16, 21], we demonstrated that serum Se levels were positively correlated with serum Cu in both T1D and T2D groups, and urinary levels of Se were positively associated with serum Zn and urinary Cu, Zn, Ca, and Mg and negatively correlated with serum Ca and Mg in T2D group.

A recent study showed low serum Zn was an independent risk factor for anemia in children and mediated the effect of low Se on Hb [22]. That explained why urinary Se is positively correlated with RBC and Hb in patients with T2D in the present study. To our knowledge, we demonstrated for the first time that urinary levels of Se were positively correlated with urinary Zn and Mg both in DR and DPN groups.

### Effect of Simvastatin Treatment on Serum or Urinary Se Levels

In the present study, 1 month of simvastatin therapy reduced serum Se levels, but had no effects on urinary Se levels. A recent study showed statins inhibit the biosynthesis of selenium-containing proteins, one of which is glutathione peroxidase serving to suppress peroxidative stress [23]. An impairment of selenoprotein biosynthesis may be a factor in congestive heart failure, reminiscent of the dilated cardiomyopathies seen with selenium deficiency [23]. In HIV-infected adults with rosuvastatin treatment, over 48 weeks, Se concentrations increased significantly in the rosuvastatin group [24]. The reason for the inconsistency between our study and this study may be due to the different duration of treatment with statin.

There are some limitations: (1) the sample size is quite small; (2) we did not collect 24-h urine volume for better evaluation of renal function; (3) as for lipid control drug, the duration is quite short and may lead to non-significant results.

## Conclusions

The effect of Se on the prevalence of diabetes has received a great attention. The present study investigated the serum and urinary Se status in patients with the pre-diabetes and diabetes in northeast China. Compared to previous paper, we found that (1) the serum Se level was dramatically lower in patients with T1D and was significantly higher in IFG subjects, and the urinary Se concentration was markedly lower in IGT and T2D groups, while urinary Se levels were all significantly lower in T2D patients with DN, DR, or DPN than uncomplicated T2D subjects. (2) The serum Se levels were positively correlated with serum Zn in both IFG and IGT groups, while urinary Se were positively associated with urinary Zn and Cu in IGT group. The serum Se levels were positively correlated with serum Cu in both T1D and T2D groups, and urinary levels of Se were positively associated with serum Zn and urinary Cu, Zn, Ca, and Mg and negatively correlated with serum Ca and Mg in T2D group, while the urinary levels of Se were positively correlated with urinary Zn and Mg both in DR and DPN groups. (3) One month of simvastatin therapy reduced serum Se levels, but had no effects on urinary Se levels.

Therefore, the potential role of Se in diabetes should receive attention. Reasonable Se supplementation in diabetic patients should be further explored.
